# Anti-Prion Drug mPPIg5 Inhibits PrP^C^ Conversion to PrP^Sc^

**DOI:** 10.1371/journal.pone.0055282

**Published:** 2013-01-28

**Authors:** James M. McCarthy, Markus Franke, Ulrike K. Resenberger, Sibeal Waldron, Jeremy C. Simpson, Jörg Tatzelt, Dietmar Appelhans, Mark S. Rogers

**Affiliations:** 1 School of Biology and Environmental Science, University College Dublin, Belfield, Dublin, Ireland; 2 Leibniz Institute of Polymer Research Dresden, Dresden, Germany; 3 Neurobiochemistry, Adolf-Butenandt-Institute, Ludwig-Maximilians-University Munich, Munich, Germany; 4 German Center for Neurodegenerative Diseases (DZNE), Munich, Germany; Deutsches Zentrum für Neurodegenerative Erkrankungen e.V., Germany

## Abstract

Prion diseases, also known as transmissible spongiform encephalopathies, are a group of fatal neurodegenerative diseases that include scrapie in sheep, bovine spongiform encephalopathy (BSE) in cattle and Creutzfeldt-Jakob disease (CJD) in humans. The ‘protein only hypothesis’ advocates that PrP^Sc^, an abnormal isoform of the cellular protein PrP^C^, is the main and possibly sole component of prion infectious agents. Currently, no effective therapy exists for these diseases at the symptomatic phase for either humans or animals, though a number of compounds have demonstrated the ability to eliminate PrPSc in cell culture models. Of particular interest are synthetic polymers known as dendrimers which possess the unique ability to eliminate PrP^Sc^ in both an intracellular and *in vitro* setting. The efficacy and mode of action of the novel anti-prion dendrimer mPPIg5 was investigated through the creation of a number of innovative bio-assays based upon the scrapie cell assay. These assays were used to demonstrate that mPPIg5 is a highly effective anti-prion drug which acts, at least in part, through the inhibition of PrP^C^ to PrP^Sc^ conversion. Understanding how a drug works is a vital component in maximising its performance. By establishing the efficacy and method of action of mPPIg5, this study will help determine which drugs are most likely to enhance this effect and also aid the design of dendrimers with anti-prion capabilities for the future.

## Introduction

Prion diseases are a group of fatal neurodegenerative diseases that can be genetic, infectious or sporadic in origin. They include scrapie in sheep, bovine spongiform encephalopathy (BSE) in cattle, and Creutzfeldt-Jakob disease (CJD) in humans. The ‘protein only hypothesis’ advocates that PrP^Sc^, a misfolded form of the endogenous prion protein, is the main and possibly sole component of prion infectious agents [1], [2]. No effective therapy exists for prion diseases at the symptomatic phase for either humans or animals. However, several molecules including congo red [3], sulphated polyanions [4], cyclic tetrapyrroles (porphyrins and phthalocyanines) [5], branched polyamines [6], [7], suramin [8], STI571 [9] and quinacrine [10] have demonstrated the ability to eliminate PrP^Sc^ in cell culture models. Synthetic macromolecules known as dendrimers are a particularly interesting class of anti-prion compounds as numerous dendrimer species possess the ability to eliminate PrP^Sc^ in both an intracellular and *in vitro* setting. Evidence suggests that this is achieved by interacting directly with PrP^Sc^ and rendering it sensitive to proteases [6], [7]. Within the cell this is thought to occur within acidic endosomes or lysosomes [6] though recently it has been speculated that certain dendrimers may eliminate PrP^Sc^ by inhibiting the conversion of PrP^C^ to PrP^Sc^ [11], [12].

**Figure 1:**
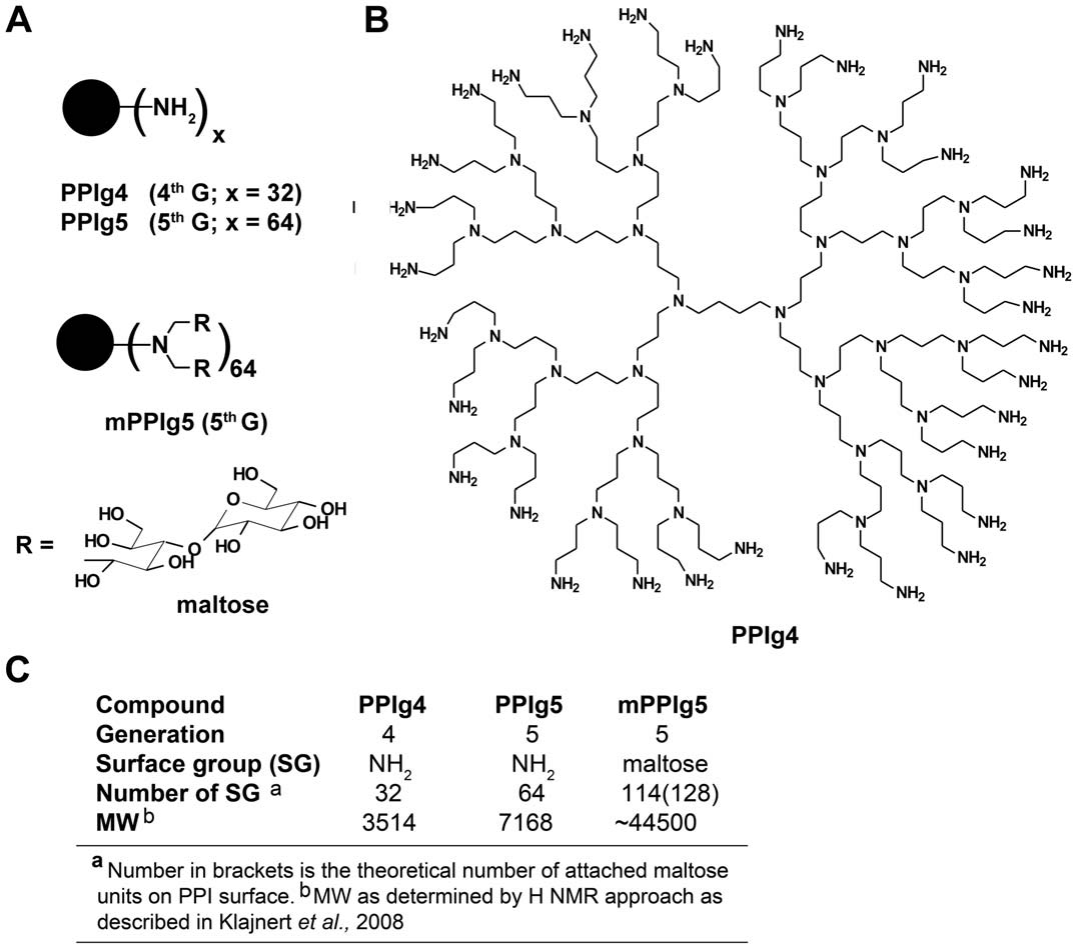
Schematic description of dendrimers PPIg4, PPIg5 and mPPIg5 used in this study. **A**) mPPIg5 is obtained by reductive amination of PPIg5 [13], [14]. **B**) Molecular structure of cationic 4th generation poly(propylene imine) (PPIg4) with the smallest size used in biological experiments. **C**) Molecular weights for the dendrimers utilised in this study.

In the present work, we examined the anti-prion efficacy of maltose modified poly(propylene imine) generation five dendrimers (mPPIg5) (Figure 1) and investigated their mode of action. This new class of branched-polyamines differs from dendrimers previously reported to possess anti-prion activity as they possess neutral rather than cationic surface groups due to the presence of maltose in their outer shell [13]. They exhibit a markedly reduced toxicity over the more established cationic dendrimers yet still retain their anti-prion activity [14].

The efficacy of an anti-prion drug in a cell culture system is generally assessed by monitoring the elimination of protease resistant PrP^Sc^ over time through the use of PK digestion (to remove PrP^C^) and immunoblotting (to detect the remaining protease resistant PrP^Sc^). As western blotting is quite cumbersome to perform and, at best, a semi-quantitative technique, we saw the potential to develop the highly quantitative and high throughput scrapie cell assay as a method of measuring the efficacy of anti-prion drugs. The standard scrapie cell assay (sSCA) is an ELISA spot assay and an alternative to the animal bioassay for the detection of prions [15]. In the sSCA, susceptible cells are exposed for 4 days to a sample which potentially harbours infectious prions. If prions are within this sample they will infect the cells and propagate over time to detectable levels. The cells are then attached to a Multiscreen 96 well plate and undergo numerous treatments including a protease digestion step in which PrP^C^ is removed and an immuno-detection step where protease resistant PrP^Sc^ is detected [15]. Infected cells are visualised as spots on the Multiscreen plate membrane using a microscope and camera system for quantification. We used the ELISA spot portion of this system in reverse to monitor the decrease in PrP^Sc^ producing cells over time following the application of mPPIg5. To achieve this, the protease digestion step of the sSCA was altered to produce an assay more suitable for detecting the decrease in prion infected cells following treatment with a therapeutic agent. The modified assay, called the modified SCA (mSCA), detects all forms of protease resistant PrP^Sc^ (Table 1)_._

**Table 1:** Summary of the standard scrapie cell assay and variants developed in this study.

Assay	Standard SCA	Modified SCA	N-terminal specific
	(sSCA)	(mSCA)	SCA (nSCA)
**Protease**	PK in lysis buffer	Trypsin in PBS	Trypsin in PBS
	**SAF83**	**SAF83**	**SAF32**
**Antibody**	Recognises an epitope (aa 126–164) in central region PrP	Recognises an epitope (aa 126–164) in central region PrP	(Recognises an epitope (codon 58–89) in the octarepeat region of the N terminal of PrP)
	All protease resistant PrP^Sc^	All protease resistant PrP^Sc^	Protease resistant FL PrP^Sc^ only
**PrP**			
**isoform**	- PrP^27–30^	- PrP^27–30^	
**detected**	- FL PrP^Sc^ (converted to PrP^27–30^ by PK)	- FL PrP^Sc^	

To determine how mPPIg5 was eliminating PrP^Sc^ from cells, we exploited the unique intracellular processing of the PrP^Sc^ protein. In prion infected cells, PrP^C^ encounters PrP^Sc^ at an unidentified region sometime after reaching the plasma membrane and is converted to the misfolded isoform. About 1 hour after this event residues 23–88 at the N-terminus of PrP^Sc^ are removed by a proteolytic process that is believed to occur in an acidic endosome or lysosome [16]–[18]. The truncated PrP^Sc^ formed by this event is known as PrP^27–30^. PrP^27–30^ accumulates in lysosomes and has a half-life of ≥24 hours [9], [17]. For the sake of clarity, in this study non-truncated protease resistant PrP^Sc^ will be referred to as Full Length PrP^Sc^ (FL PrP^Sc^) and truncated protease resistant PrP^Sc^ will be referred to as PrP^27–30^.

In summary: PrP^C^ →FL PrP^Sc^→PrP^27–30^.

As FL PrP^Sc^ is short lived, its intracellular level is closely related to its production rate. If the synthesis of FL PrP^Sc^ is interfered with, the level of detectable FL PrP^Sc^ drops rapidly. Thus the level of FL PrP^Sc^ can be seen as a marker of the conversion of PrP^C^ to misfolded forms. There are two basic mechanisms by which a drug can eliminate prions from a cell: (A) by increasing the degradation of pre-existing PrP^Sc^ or (B) by inhibiting the production of prions by blocking the conversion of PrP^C^ to FL PrP^Sc^. If a drug inhibits the conversion of PrP^C^ to FL PrP^Sc^, a rapid drop in FL PrP^Sc^ should occur. Thus, by monitoring the level of FL PrP^Sc^ we hoped to gain an insight into the mechanism by which mPPIg5 cleared PrP^Sc^ from cells.

A variation of the SCA, called the N terminal specific scrapie cell assay (nSCA), was created to quantify FL PrP^Sc^. This assay was based on an assay previously developed by our laboratory to detect PrP^Sc^ in its nascent state [19]. The nSCA specifically detects FL PrP^Sc^. It does not detect the truncated form (PrP^27–30^) which represents the majority of PrP^Sc^ in the cell [20]. The nSCA uses the protease trypsin to enable the detection of FL-PrP^Sc^. Trypsin digests all PrP^C^ and cleaves protease resistant PrP^Sc^ at positions 23, 24, 37 and 48. Although these are N-terminal cleavage sites, the majority of the N terminal including the Ocatpeptide Repeat region (OR) is left intact [21]. An antibody (SAF32) directed against an epitope in the OR of the N-terminal can be used to detect FL PrP^Sc^ [19]. This epitope is not present on PrP^27–30^ as the N terminal region is cleaved in the formation of PrP^27–30^ [16], [18]. Therefore, the use of an N terminal antibody to differentiate between FL PrP^Sc^ and PrP^27–30^, and trypsin to remove PrP^C^ but leave the majority of the N terminal of FL PrP^Sc^ intact, enables the nSCA to directly identify FL PrP^Sc^ present in a cell (Table 1). The sSCA is not capable of this as the PK used in this assay cleaves the N terminus of FL PrP^Sc^ reducing it to PrP^27–30^ (comparable to the cleavage that occurs in lysosomes). Therefore in the sSCA, FL PrP^Sc^ and PrP^27–30^ cannot be differentiated.

The ability to detect and quantify protease resistant PrP^Sc^ and FL PrP^Sc^ provides a powerful approach in assessing the efficacy and method of action of current and future therapeutics in the treatment of prion diseases. In this study the mSCA and nSCA were used to investigate the efficacy and mode of action of the anti-prion therapeutic mPPIg5. The assays demonstrated that mPPIg5 is a highly effective anti-prion drug which acts, at least in part, through the inhibition of PrP^C^ to PrP^Sc^ conversion in 22L infected N2a cells.

## Materials and Methods

### Reagents and therapeutic compounds

All reagents used in this study were analytical grade, and unless otherwise stated, obtained from Sigma. The poly(propylene imine) generation four (PPIg4; DAB-Am32) and generation five (PPIg5; DAB-Am64) dendrimers were obtained from SyMO-Chem (Eindhoven, Netherland). PPIg5 dendrimers modified with a dense maltose shell (mPPIg5; Mw 44,500 g/mol) were prepared by a reductive amination of PPIg5 in the presence of maltose as described previously [13], [14]. Dendrimer stock compounds were prepared as 20 mg/ml solutions in sterile H_2_O. Suramin was prepared as a 200 mg/ml stock solution in 0.9% NaCl in dH_2_O. STI571 (Stratech Scientific) was prepared as a 10 mM stock solution in dimethyl sulfoxide. NH_4_Cl was prepared as a 1M stock solution in Dulbecco's Modified Eagle Medium (DMEM)(Gibco). All stock solutions were sterilized by filtration through a 0.22 µm syringe filter (Millipore).

### Cell culture and treatment

N2a cells were kindly donated by Dr. Byron Caughey (Rocky Mountain Laboratories, National Institutes of Health, Hamilton, MT, USA) [22]. These were subcloned and infected with the murine adapted scrapie prion strain RML by the method of Bosque and Prusiner [23]. N2a#58 cells and N2a#58 cells infected with the mouse adapted prion strain 22L (22LN2a#58), were kindly donated by Dr Sylvain Lehmann (Institut de Génétique Humaine du CNRS, Montpellier, France). N2a#58 cells are N2a cells transfected with mouse PrP^a/a^ and produce ∼6x the normal levels of PrP^C^ [24]. All cell lines were maintained as monolayers at 37°C in 8% CO_2_ in cultivation media consisting of Dulbecco's Modified Eagle Medium (DMEM)(Gibco) supplemented with 2 mM glutamine, 100U/ml penicillin, 100 μg/ml streptomycin and 10% heat inactivated foetal bovine serum (FBS)(Gibco). In addition, the transfected N2a#58 cells were treated with 300 g/ml G418. The medium for all cell lines was replaced every 2–3 days and cells typically reached confluence after 4–5 days at which stage they were split for subculture. N2a and ScN2a used for metabolic labelling were cultured as described earlier [25]. For treatment of cells with various therapeutics, cells were generally sub-passaged 24 hours prior to treatment at a dilution that allowed cells to be confluent at the end of treatment. Suramin, STI571, mPPIg5 and NH_4_Cl were diluted from stock solutions in regular culture media to the stated concentrations.

### Scrapie Cell Assay

The ELISA portion of the Scrapie cell assay (SCA) was adapted from the protocol previously published by Klohn *et al* [15] and all steps were carried out at room temperature unless otherwise stated. The ELISPOT plate (MultiscreenHTS 96 well filtration plates with Immobilon-P transfer membrane, 0.45 µm; Millipore) was activated with 60 µl 70% methanol per well. The Methanol was removed by vacuum (MultiScreen vacuum; Millipore), wells washed twice with 160 µl PBS and 60 µl PBS added to each well to prevent drying out. Cells to be examined were washed twice with PBS and suspended in DMEM by gentle pipetting. Cells were diluted to 100,000 or 25,000 cells per ml depending on experiment and 200 µl of this dilution added to each well to give 20,000 or 5,000 cells per well. Cells were attached to the membrane by vacuum and the elispot plate placed in an incubator at 60°C for 1 hour without its lid. After 1 hour, the plate was either wrapped in cling film and stored at 4°C for up to two weeks or directly treated with protease.

Protease treatment varied depending on the experiment. For the standard scrapie cell assay 60 µl of PK (0.3 µg/ml for 5000 cells; 0.5 µg/ml for 20,000) diluted in lysis buffer (50 mM Tris HCl pH 8.0, 150 mM NaCl, 0.5% sodium deoxycholate, 0.5% Triton X-100) was used. For the modified and N terminal specific scrapie cell assay, 60 µl of trypsin (10 µg/ml for 5000 cells; 15 µg/ml for 20,000 cells) diluted in PBS (pH7.4) was utilised. Protease treatment was carried out at 37°C and 80rpm for one hour, followed by one wash with 160 µl PBS. The reaction was stopped by addition of 150 µl 2 mM PMSF (in PBS, pH 7.4) for 10 minutes. Cells were washed twice with 160 µl PBS, pH 7.4. and denatured by treatment with 120 µl 3M guanidinium thiocyanate (GSCN), 10mM Tris-HCl (pH 8) for 10 minutes. Following this wells were washed four times with PBS, pH 7.4 and incubated with 160 µl of Superblock (Pierce) for 1 hour. The superblock was removed and 60 µl primary antibody in TBS-T/1% non-fat milk powder (Marvel) added at the appropriate dilution for 1 hour – For the sSCA and mSCA, SAF83 was used at a final concentration of 20ng/ml. For the nSCA, SAF32 was employed at a final concentration of 266ng/ml. The wells were washed seven times with 160 µl TBS-T and incubated with 60µl ALP-conjugated anti-mouse secondary antibody (0.1 µg/ml in TBS-T/1% non-fat milk powder) for 1 hour. The wells were washed eight times with TBS-T and signal developed by incubation with 60 µl/well BCIP for 16 minutes in the dark. The development was stopped by two washes with 160 µl dH_2_O. The plastic casing at the back of the elispot plate was then removed and the wells washed a further two times with 160 µl dH_2_O. PrP^Sc^ positive cells were detected and quantified using the Zeiss Elispot software system and Zeiss AX-10 imager (Zeiss).

### Lysate preparation

For analysis of intracellular protein content, confluent cells were washed twice with PBS, pH 7.4 and lysed with ice cold lysis buffer (10 mM Tris, pH 7.4, 100 mM NaCl, 10 mM EDTA, 0.5% deoxycholic acid, 0.5% Triton X-100) for 5 minutes. Cells were detached from plate by pipetting and centrifuged at 1,000×g for 5 minutes to remove cellular debris. Protein concentration of the lysate was measured by BCA assay (Pierce) according to the manufacturer's instructions. For lysis of cells in 4% sarkosyl in PBS (pH 7.4), the same procedure as above was initially followed but lysis was performed with 4% sarkosyl in PBS (pH 7.4), not cellular lysis buffer. The lysate was subsequently homogenised by successive passage through 20, and 23 gauge needles. Protein concentration of the lysate was measured by BCA and the cell lysates were stored at −20°C and thawed on ice before use. For dendrimer treatment of lysates (lysate in 4% sarkosyl only) a final concentration of 2.4 mg/ml mPPIg5 was incubated with samples for 3 hours at 37°C and 450rpm. Protease resistant PrP^Sc^ content of all lysates was analysed by proteinase K (PK) treatment for 30 minutes at 37°C at a ratio of 250:1 using an amount of 100 µg cellular lysate (total protein) per sample. Reactions were stopped by addition of 5 mM PMSF. The sample was centrifuged at 16,000 g for 30 minutes, supernatant discarded and pellet re-suspended in 1× SDS sample buffer for immunoblot analysis.

### Immunoblot Analysis

Protein samples in 1× SDS sample buffer were incubated for 5 minutes at 95°C prior to electrophoresis on a 12.5% SDS polyacrylamide gel. After transfer (100 mV, 1 h) to a PVDF membrane (Millipore) and rinsing in TBST (TBS-0.5% Tween 20), unspecific binding sites were blocked by incubation in 5% dried milk powder (Marvel) in TBST for 1 h, with shaking (250 rpm). The membrane was washed five times in TBST (5 minutes each, shaking at 250 rpm), and incubated with SAF83 (SPI Bio, diluted to 20 ng/mL in TBST) overnight. The membrane was washed 5 times and exposed to goat anti-mouse IgG-ALP secondary antibody (Promega, diluted to 0.1 µg/ml in TBST) for 1.5 hours, followed by five additional washing steps in TBST (5 minutes each, shaking at 250 rpm). Blots were developed by exposure to 10 mls 5-Bromo-4-chloro-3-indolyl phosphate (BCIP) for 5 minutes, followed by two washes with dH_2_O. Developed membranes were digitized (imaged) using the Fujifilm LAS 4000.

### Purification of PrP^27–30^ from prion infected N2a cells (ScN2a)

ScN2a cells were grown to confluence on 100 mm plates and washed twice with 4mls PBS (pH 7.4). Cells were lysed with ice cold 600 µl low detergent lysis buffer (10mM Tris, pH 7.4, 100 mM NaCl, 0.05% deoxycholic acid, 0.05% Triton X-100) and centrifuged at 1,000 g for 5 mins to remove cellular debris. Protein concentration of this postnuclear supernatant was measured by BCA (Pierce). The lysate was treated with proteinase K (PK) at a ratio of 1:50, protease to protein, for 2 hours at 37°C. The reaction was stopped by the addition of 5 mM PMSF (1/20 dilution of 100 mM PMSF). 500 µg aliquots of the lysate were transferred into 1.5 ml eppendorfs and centrifuged at 16,000g for 30 minutes. The supernatant was discarded and the pellet washed twice in 100 ml PBS (pH 7.4) by centrifugation at 16,000 g for 4 minutes. The resulting pellet was re-suspended in 50 µl PBS (pH 7.4) to give a final concentration of 10 µg/µl (before PK).

### Inoculation of N2a cells with PrP^27–30^

N2a cells were seeded in a 96 well dish (for infection study) or 24 well dish (for nSCA validation) at a ratio of 20,000 and 150,000 cells per well respectively. Cells were incubated overnight in regular culture media at 37°C, 8% CO_2_. PrP^27–30^ purified from ScN2a cells was diluted to various degrees in cultivation media and added to the appropriate well. Cells were grown for 96 hours at 37°C and 8% CO_2_. The inoculated cells were either examined after 1 hour (nSCA validation) or cultured for four days before passaging at a 1/4 dilution, then cells cultured to confluence and passaged a further 3 times at 1/10 dilution before analysis by mSCA (Infection study).

### Confocal Microscopy

Coverslips (13mm; Agar Scientific) were sterilised in 70% IMS and incubated overnight in Poly-D-Lysine (50 µg/ml). Following a rinse in sterile dH_2_O, coverslips were placed at the bottom of 6 well TC plates. N2a and N2a#58 cells were added at a dilution of 30,000 cells/well in cultivation media and let adhere to coverslips for 24 hours. After 24 hours, media was removed and cultivation media with either suramin (200 µg/ml) or mPPIg5 (20 µg/ml) added. Mock treated cells were treated with cultivation media +10 µl sterile dH_2_O. After 48 hours cells were fixed in 3% Paraformaldehyde for 20 minutes, quenched with 30 mM Glycine in PBS (pH 7.5) for 5 minutes and washed three times with PBS (pH 7.4) in 6 well TC plates. Cells to be permeabilized were treated with 0.1% Triton X-100 in PBS (pH 7.4) for 5 minutes and washed three times with PBS (pH 7.4). Primary antibody was applied for one hour (50 µl SAF32, 500 ng/ml in PBS). Coverslips were washed three times with PBS and 50 µl Alexa-488 conjugated anti-mouse secondary antibody (5 µg/ml in PBS; Invitrogen) applied. Following this, coverslips were washed once with PBS, treated for 3mins with the nuclear stain Hoechst 33342 (1:5000 in PBS; Invitrogen), and washed twice with PBS. Coverslips were mounted on glass slides using Mowiol and stored in the dark for 24 hours. Immunostained cells on coverslips were analysed by confocal microscopy using the Olympus FV1000 confocal microscope (Olympus). A 60x 1.35NA UPL SAPO objective was used. All images were acquired equally; 2x zoom, a scanning speed of 10.0 µs/pixel, with 3x kalman line averaging and sequential acquisition.

### PIPLC analysis of cells

N2a#58 cells were seeded onto 60 mm plates and cultured for 24 hours. After 24 hours the media was removed, cells washed once in PBS (pH 7.4) and treatment media added for 48 hours. Treatment consisted of cultivation media with suramin (200 µg/ml), mPPIg5 (20 µg/ml) or sterile dH_2_O (10 µl) for mock treatment. After 48 hours media was removed and cells were mock treated with serum free DMEM at 37°C or treated for 2 hours with 0.5U/ml PIPLC in serum free DMEM. The media from all plates was collected and cells washed three times with PBS (pH 7.4) before being lysed as described previously. The protein in the media was ethanol precipitated and the PrP^C^ content of this and the lysed cells (25 µg) determined by immunoblotting.

### Metabolic labelling

Metabolic labelling was carried out as described previously [26]. Briefly, N2a cells cultivated in 3.5 cm dishes were transfected with 1 µg DNA by a liposome-mediated method using LipofectAMINE Plus reagent (Invitrogen) according to the manufacturer's instruction. The mouse PrP construct used contain a 3F4 tag [25]. Cells were starved for 30 minutes in methionine-free modified Eaglés medium (Invitrogen) and subsequently labelled for 1 hour with 300 µCi/ml L-[^35^S] methionine (Hartmann Analytics; >37 TBq/mmol) in methionine-free modified Eaglés medium (Invitrogen). For the chase, the labelling medium was removed, and cells were washed twice and incubated in complete medium for 4 hours. When indicated, maltose modified Poly(propylene imine) generation 5 (mPPIg5) was present during the starving, labelling and chase periods (final concentration 20 µg/ml). PrP was analysed by immunoprecipitation with the monoclonal 3F4 antibody [25], [27]. The immunopellet was analysed by SDS-PAGE and autoradiography [28].

## Results

### Efficacy of mPPIg5

**Figure 2:**
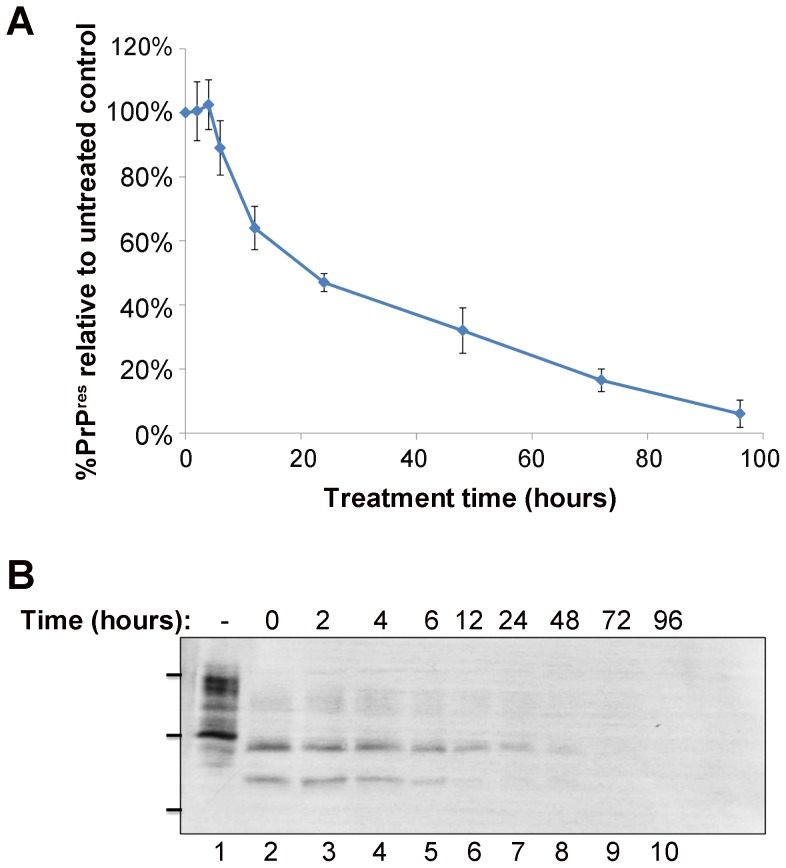
mPPIg5 treatment of 22LN2a#58 cells . **A**) 22LN2a#58 cells were either treated with 20 µg/ml mPPIg5 or mock-treated for increasing lengths of time before analysis of cells for PrP^Sc^ content via mSCA. The % difference in PrP^Sc^ content between mPPIg5 and mock treated cells at each time point was calculated and the decrease in PrP^Sc^ over time plotted. Error bars represent SD; n = 3. **B**) 22LN2a#58 cells were treated with 20 µg/ml mPPIg5 for increasing lengths of time before detection of protease resistant PrP^Sc^ via PK digestion and immunoblotting (lanes 3–10). Samples in lane 2 were not treated whilst samples in lane 1 were not treated or PK digested. Apparent molecular mass based on migration of protein standards is indicated for 17, 25, and 30 kDa. n = 3.

The modified scrapie cell assay (mSCA) was developed in this study and is used to quantify the decrease in PrP^Sc^ producing cells over time following treatment with a therapeutic. In comparison to the standard scrapie cell assay (sSCA) which uses PK in lysis buffer, the mSCA employs trypsin in PBS as the protease digestion step necessary to remove PrP^C^. This produces more intense and compact cells which are easier to quantify and results in an assay better able to detect a decrease in PrP^Sc^ producing cells over time (Figure S1). The mSCA was used to investigate the efficacy of maltose modified PPI dendrimers (mPPIg5) in curing prion infected cells. mPPIg5 has previously been shown to eliminate PrP^Sc^ from RML infected N2a cells (ScN2a) [14]. To examine the ability of mPPIg5 to cure cells infected with strains other than RML, 22L infected N2a#58 cells (22LN2a#58) were treated with 20 µg/ml mPPIg5 for increasing periods of time and cells assessed via the modified scrapie cell assay (mSCA) (Figure 2A). The mSCA illustrates that mPPIg5 eliminates protease resistant PrP^Sc^ from 22LN2a#58 cells in a highly efficient manner and highlights the promise of modified dendrimers as anti-prion therapeutics. The traditional method of PrP^Sc^ detection – PK digestion and immunoblot – was used to confirm the result (Figure 2B). The optimum treatment concentration of mPPIg5 was also determined using the mSCA (Figure S2) and found to be in agreement with the literature at 10–20 µg/ml [14].

### Intracellular method of action of mPPIg5

**Figure 3:**
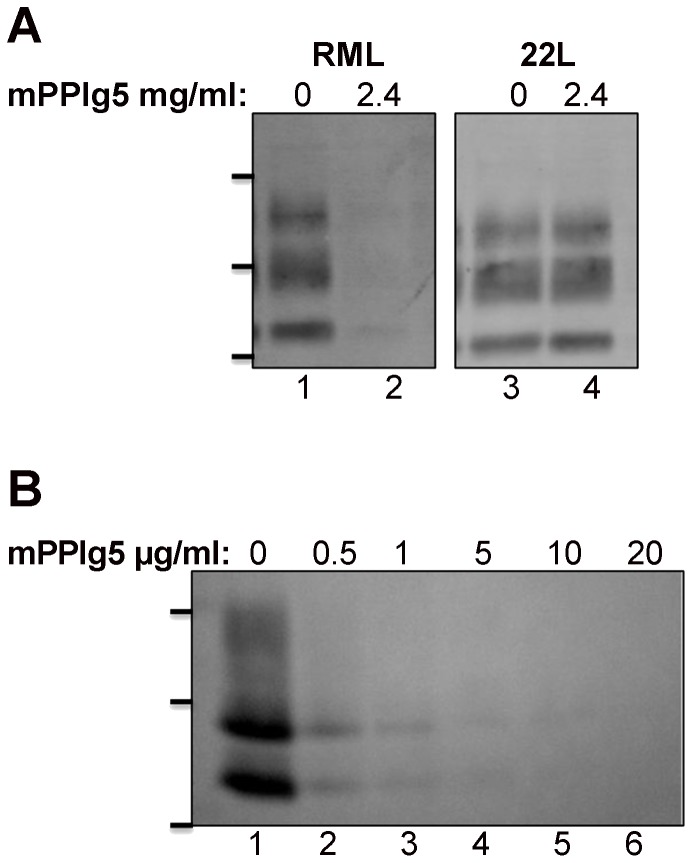
mPPIg5 treatment of ScN2a cells and treatment of lysates from RML and 22L infected N2a cells. **A**) RML infected N2a cells and 22L infected N2a#58 cells were lysed in 4% sarkosyl (in PBS pH 7.4). 100 µg of lysate was then left untreated or treated with 2.4 mg/ml of mPPIg5 dendrimers for 3 hours before analysis for protease resistant PrP^Sc^ content by PK digestion and immunoblot. **B**) RML infected N2a cells were treated with increasing concentrations of mPPIg5 for 4 days before analysis by PK digest and immunoblot for PrP^Sc^. Apparent molecular mass based on migration of protein standards is indicated for 17, 25, and 30 kDa in both panel A and B. n = 3.

Dendrimers are believed to achieve their anti-prion effect by acting directly on PrP^Sc^ within lysosomes and rendering them sensitive to proteases [6]. The ability of dendrimers to render PrP^Sc^ from brain homogenate protease sensitive *in vitro* supports this [6], [7]. mPPIg5 eliminated protease resistant PrP^Sc^ from 22L infected N2a#58 cells (Figure 2), so it was expected that mPPIg5 would also be effective at eliminating protease resistant PrP^Sc^ from lysates derived from 22L infected N2a#58 cells. However, whilst mPPIg5 was able to eliminate protease resistant PrP^Sc^ from lysates derived from RML infected cells *in vitro,* it had no effect on lysates derived from 22L infected cells (Figure 3A). In contrast, intracellular treatment with mPPIg5 eliminated protease resistant PrP^Sc^ from both RML (Figure 3B) and 22L infected cells (Figure 2). This suggested that the method by which mPPIg5 eliminated PrP^Sc^ intracellularly and *in vitro* may be different – mPPIg5 dendrimers may not render PrP^Sc^ protease sensitive *in vivo;* the presumed mode of action for dendrimers. They may interfere with the PrP^C^ → PrP^Sc^ conversion process. This possibility was investigated by developing a variation of the SCA capable of quantifying Full Length PrP^Sc^ (FL PrP^Sc^) called the N terminal specific SCA (nSCA).

**Figure 4:**
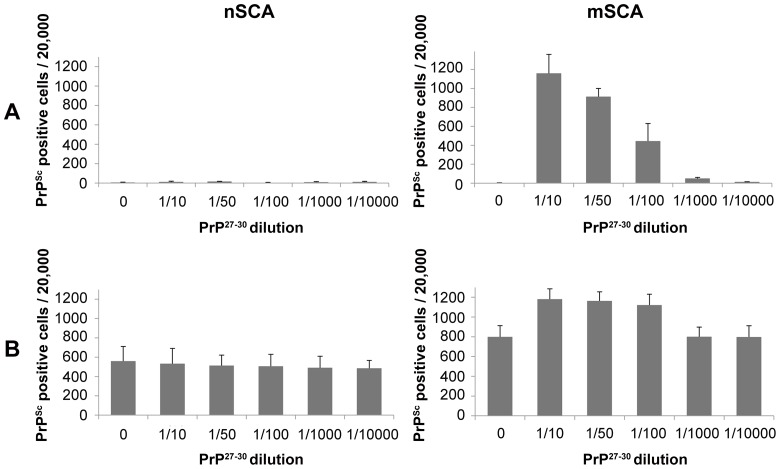
Validation of nSCA. **A**) N2a cells and **B**) ScN2a cells were inoculated for 1.5 hours with increasing dilutions of PrP^27–30^. The infective media was removed and replaced with regular DMEM for an additional 3 hours before cells were repeatedly washed with PBS and 20,000 cells attached per well of an ELISPOT plate. Cells were examined via the nSCA and mSCA. Positive result is defined as >30 spots/20,000 cells. The software used to count spots (infected cells) cannot count accurately above 1000 spots so a dose response curve is not observed with the mSCA for the ScN2a cells inoculated with PrP^27–30^. Error bars represent SD; n = 2.

The nSCA was created to directly quantify FL PrP^Sc^. To validate this assay and confirm that it only detects FL PrP^Sc^ and not PrP^C^ or PrP^27–30^, N2a cells were inoculated with exogenous PrP^27–30^ for 1 hour and assayed by the mSCA and nSCA (Figure 4A). (For details on production and validation of PrP^27–30^ see Fig S3). The nSCA did not detect PrP^27–30^. The mSCA (which can detect PrP^27–30^) acted as a positive control to show that PrP^27–30^ was associated with the cells and illustrated a dose response curve matching the dilutions of inoculates used (Figure 4A).

To demonstrate that the nSCA can detect FL PrP^Sc^, RML infected N2a cells (ScN2a) were inoculated with PrP^27-30^ and nSCA and mSCA performed (Figure 4B). The nSCA detected the endogenous FL PrP^Sc^ within the ScN2a cells but not the additional exogenous PrP^27–30^. The mSCA detected both the endogenous FL PrP^Sc^ and the exogenous and endogenous PrP^27–30^. These results demonstrate the nSCA's ability to differentiate between FL PrP^Sc^ and PrP^27–30^ in prion infected cells. Having demonstrated that the nSCA can selectively detect FL PrP^Sc^, we sought to determine if the nSCA could be used to deduce mPPIg5′s intracellular mode of action.

The hypothesis was:

1. If mPPIg5 interfered with the conversion of PrP^C^ to FL PrP^Sc^, a rapid decrease in FL PrP^Sc^ levels would be expected; PrP^C^ to FL PrP^Sc^ conversion would be inhibited and so no new FL PrP^Sc^ would be produced. Existing FL PrP^Sc^ is naturally cleaved to PrP^27–30^ within ∼1 hour of its synthesis [16], [18] and so FL PrP^Sc^ levels would be expected to drop rapidly.
2. If mPPIg5 acted in the lysosome to increase the degradation of PrP^Sc^, no short term effect would be observed on FL PrP^Sc^; mPPIg5 may induce some degradation of FL PrP^Sc^, but PrP^C^ conversion to FL PrP^Sc^ would not be significantly perturbed and so FL PrP^Sc^ levels would not be expected to drop rapidly over the short term.

To validate this approach, two drugs with an established anti-prion mode of action were also examined. Suramin is a drug which achieves its anti-prion effect by inhibiting the conversion of PrP^C^ to PrP^Sc^. It achieves this by inhibiting the regular trafficking of PrP^C^ and causing its aggregation as non-protease resistant aggregates in a post ER/Golgi compartment. This alteration in trafficking prevents its conversion to PrP^Sc^ [8]. STI571 is a tyrosine kinase inhibitor whose effect on c-abl tyrosine kinase results in the increased lysosomal degradation of PrP^Sc^ [9]. We hypothesised that suramin would have a dramatic effect on FL PrP^Sc^ levels over the first 48 hours of treatment whilst STI571 would have a minimal effect over this time period. The mode of action of mPPIg5 could then be deduced by correlating its effect on FL PrP^Sc^ with these drugs.

**Figure 5:**
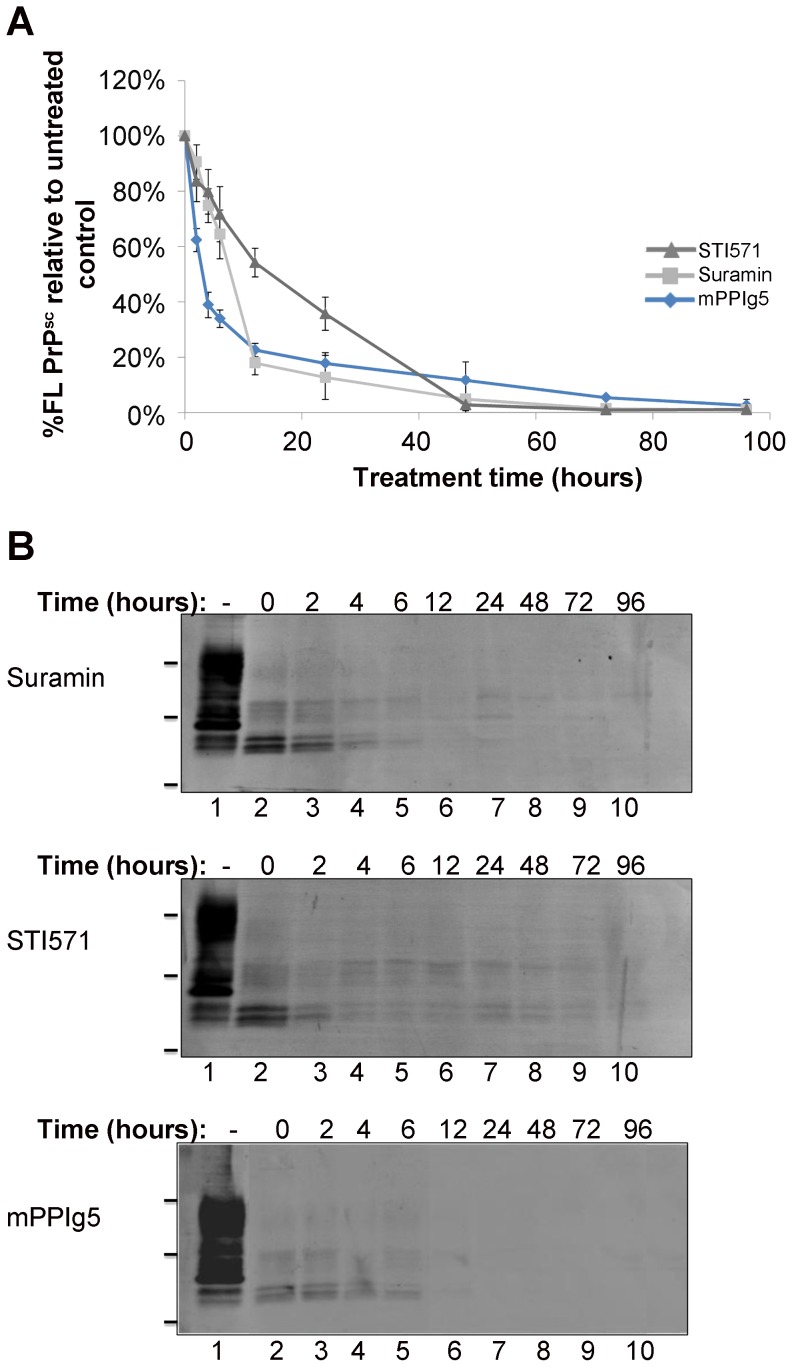
Effect of Suramin, STI571 and mPPIg5 treatment on FL PrP^Sc^ levels . **A)** 22LN2a#58 cells were treated with either STI571 (10 µm), suramin (200 µg/ml), mPPIg5 (20 µg/ml) or mock treated for increasing periods of time before analysis for FL PrP^Sc^ content by nSCA. The % difference in PrP^Sc^ content between therapeutically treated and mock treated cells at each time point was calculated for each drug (STI571, Suramin and mPPIg5) and the decrease in FL PrP^Sc^ over time plotted. Error bars represent SD; n = 3. **B**) To validate the result, 22LN2a#58 cells treated with STI571 (10 µm), suramin (200 µg/ml) and mPPIg5 (20 µg/ml) for various periods of time were also lysed and analysed for FL PrP^Sc^ content by trypsin digestion and immunoblotting with SAF32 (Lanes 3–10). Samples in lane 2 were not treated whilst samples in lane 1 were not treated or PK digested. Apparent molecular mass based on migration of protein standards is indicated for 17, 25, and 30 kDa. n = 3.

To investigate this, 22LN2a#58 cells were treated with suramin (200 µg/ml), STI571 (10 µM) and mPPIg5 (20 µg/ml) for increasing periods of time before analysis for FL PrP^Sc^ levels via the nSCA and immunoblotting (Figure 5). Both suramin and mPPIg5 treatment resulted in a rapid decrease of FL PrP^Sc^ levels with ∼80% of FL PrP^Sc^ eliminated after 12 hours. Contrary to expectations, STI571 treatment also led to a rapid decrease in FL PrP^Sc^ levels, though the decline in FL PrP^Sc^ levels was less pronounced with ∼50% eliminated after 12 hours. The rapid loss in FL PrP^Sc^ levels upon STI571 treatment was unexpected. Pulse chase experiments have demonstrated that STI571 does not interfere with the production of protease resistant PrP^Sc^ [9]. Production of protease resistant PrP^Sc^ must involve the production of FL PrP^Sc^ (PrP^C^ → FL PrP^Sc^ → PrP^27–30^). Therefore the decrease in FL PrP^Sc^ upon STI571 treatment while protease resistant PrP^Sc^ production continued unabated was puzzling. One explanation is that STI571 may increase the rate of truncation of FL PrP^Sc^. This would lead to a rapid decrease in detectable FL PrP^Sc^ without affecting the production of protease resistant PrP^Sc^. STI571 inhibits the tyrosine kinase c-Abl, which is thought to result in the increased lysosomal degradation of PrP^Sc^ [9]. Given its effect on the lysosome, the ability of STI571 to increase the N terminal truncation of FL PrP^Sc^ is therefore plausible. Although not alluded to by the authors, this scenario is also supported by examination of the original results from Ertmer *et al* (Figure 4A, lane 1 vs 3; Ertmer *et al*., 2004).

The rapid loss of FL PrP^Sc^ observed in mPPIg5 treated cells could be explained by an increased rate of FL PrP^Sc^ N terminal truncation. NH_4_Cl was used to examine this possibility. NH_4_Cl inhibits N terminal truncation of FL PrP^Sc^ by raising the pH of the acidic endosome where this cleavage occurs. NH_4_Cl does not interfere with the production of *de novo* PrP^Sc^ or the biogenesis and trafficking of PrP^C^ [16].

It was hypothesised:

1. If a compound inhibits the conversion of PrP^C^ to FL PrP^Sc^, no difference between cells treated in the presence and absence of NH_4_Cl should be observed; FL PrP^Sc^ is not being produced so NH_4_Cl induced inhibition of truncation should have no effect.
2. If an anti-prion compound solely achieves its effect by increasing the degradation of PrP^Sc^ isoforms (including FL PrP^Sc^), prion infected cells treated in the presence of NH_4_Cl should produce a higher level of FL PrP^Sc^ than cells treated in the absence of NH_4_Cl. There are at least two ways NH_4_Cl can achieve this. a) NH_4_Cl may inhibit the anti-prion action of a drug by raising the pH of the lysosome. In this instance, PrP^Sc^ isoforms (including FL PrP^Sc^) would not be degraded and so an increase in the level of FL PrP^Sc^ would be observed in cells treated in the presence of NH_4_Cl over cells treated in the absence of NH_4_Cl. b) In the presence of NH_4_Cl, an anti-prion drug may still degrade PrP^Sc^ isoforms. However NH_4_Cl treated cells will possess an increased level of FL PrP^Sc^. Cells treated with an anti-prion drug in the presence of NH_4_Cl will therefore display a higher level of FL PrP^Sc^ than cells treated in the absence of NH_4_Cl as there is more FL PrP^Sc^ to degrade.

**Figure 6:**
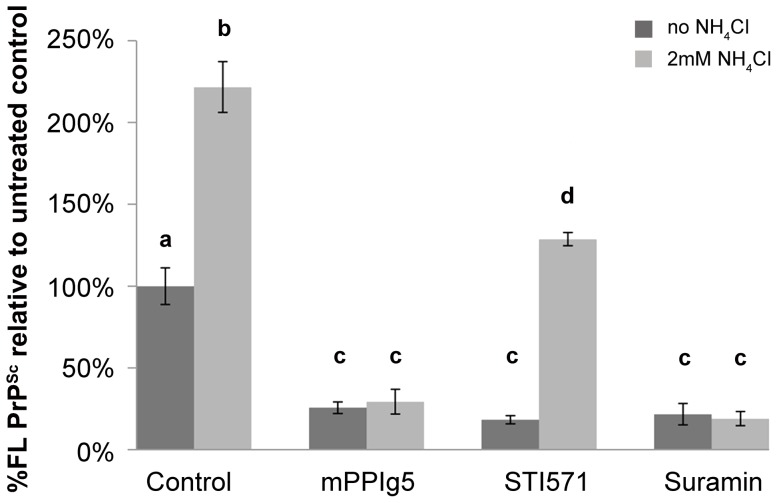
Effect of NH_4_Cl on FL PrP^Sc^ levels. 22LN2a#58 cells were mock treated or treated with mPPIg5 (20 µg/ml), STI571 (10 µM) or suramin (200 µg/ml) in the presence or the absence of 2 mM NH_4_Cl for 48 hours before analysis for FL PrP^Sc^ content via nSCA. The number of FL PrP^Sc^ containing cells in 20,000 mock treated control cells (- NH_4_Cl) is taken as the 100% value on the Y axis. All subsequent results are calculated from this. Results marked a, b, c and d are all statically significantly different from one another (p<0.001); Analysed using a Two-way ANOVA for PrP^res^, Dendrimer × NH_4_Cl (F_4,88_  = 7.99; P<0.001). Post-hoc analysis by Tukeys test; Error bars represent SD; n = 3.

To examine this hypothesis, 22LN2a#58 cells were treated for 48 hours with mPPIg5 (20 µg/ml), suramin (200 µg/ml) or STI571 (10 µM) in the presence or absence of 2 mM NH_4_Cl and analysed by nSCA (Figure 6). In the presence of NH_4_Cl, 22LN2a#58 control cells registered an increase in FL PrP^Sc^ over non NH_4_Cl treated control cells (Figure 6). This confirms that NH_4_Cl produces an increase in FL PrP^Sc^ by preventing its truncation to PrP^27–30^.

Both mPPIg5 and suramin treatment of 22LN2a#58 cells produced a decrease in FL PrP^Sc^ that was unaffected by NH_4_Cl (Figure 6). This indicates that mPPIg5 is capable of inhibiting the conversion of PrP^C^ to FL PrP^Sc^. Suramin served as a control in this experiment for a drug known to inhibit the conversion of PrP^C^ to PrP^Sc^ [8].

STI571 was used as a control for a drug known to increase the degradation of pre-existing PrP^Sc^ [9]. STI571 treatment of 22LN2a#58 cells resulted in a decrease of FL PrP^Sc^ that was partially reversed by NH_4_Cl (Figure 6). NH_4_Cl inhibition of STI571′s ability to clear PrP^Sc^ was previously reported and attributed to NH_4_Cl raising the pH of the acidic lysosome where STI571 induced degradation of PrP^Sc^ was thought to occur [9].

From this experiment we can conclude that mPPIg5 inhibits the conversion of PrP^C^ to FL PrP^Sc^. It should be noted that these results make no assumption about mPPIg5′s ability to increase the degradation of PrP^Sc^, though it does rule this out as being mPPIg'5s sole mechanism of action.

**Figure 7:**
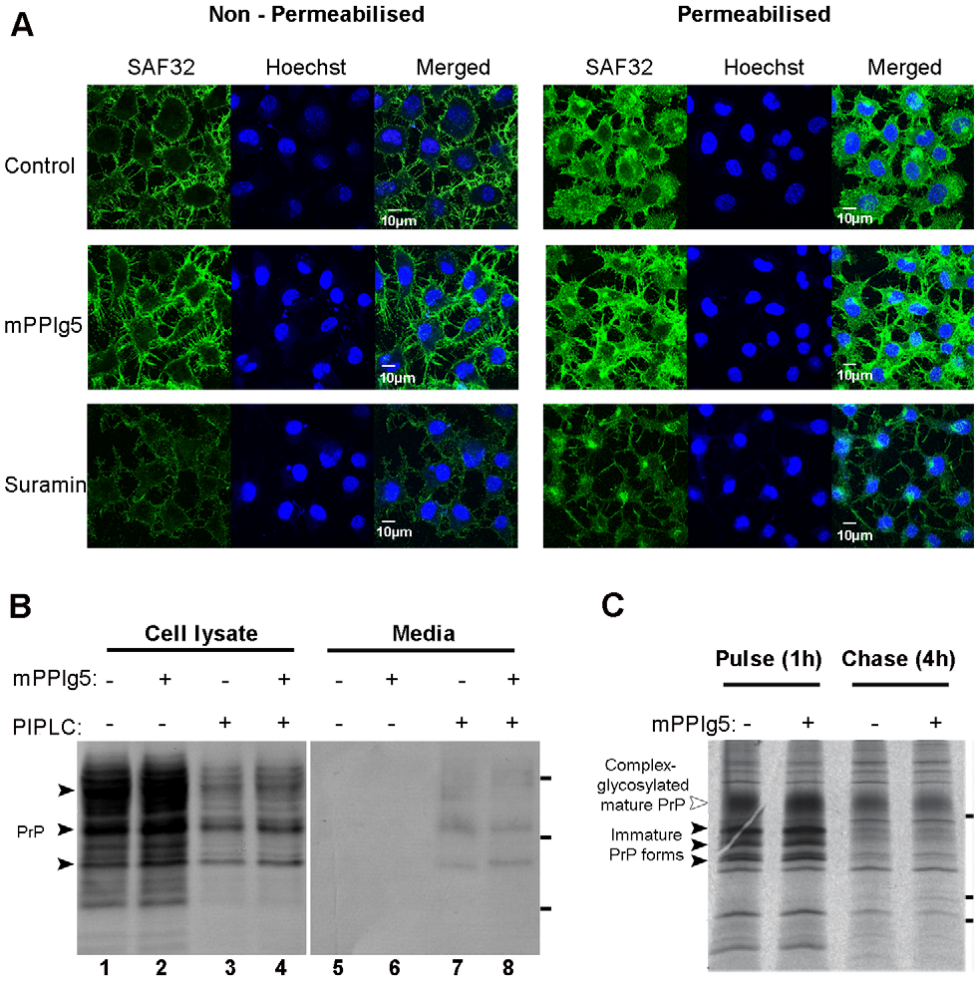
mPPIg5 effect on subcellular distribution of PrP^C^. **A**) Confocal microscopy was used to examine the cellular localization of PrP^C^. N2a#58 cells grown on coverslips were mock treated, treated with 20 µg/ml mPPIg5 or treated with 200 µg/ml suramin for 48 hours. Cells were then permeabilized or left intact before immuno-staining with SAF32 and Alexa488 secondary antibody for PrP^C^. Hoechst 33342 was used to stain DNA. All images were acquired using equal settings. n = 2. **B**) Phosphatidylinositol-specific phospholipase C (PIPLC) was used to validate the confocal microscopy result. N2a#58 cells were treated with 20 µg/ml mPPIg5 (lanes 2, 4, 6 and 8) or mock treated (lanes 1, 3, 5 and 7) for 48 hours. Cells were washed with PBS then treated for two hours with 0.5U/ml PIPLC in serum free DMEM (lanes 3, 4, 7 and 8) or mock treated with serum free DMEM (lanes 1, 2, 5 and 6). The media was collected, ethanol precipitated and examined for PrP^C^ by immunoblotting with SAF83 antibody (lanes 5–8). The remaining cells were lysed and a portion examined for PrP^C^ by immunoblot (lanes 1–4). Apparent molecular mass based on migration of protein standards is indicated for 17, 25, and 30 kDa. **C**) Biosynthesis of PrP^C^ was examined by metabolic labelling. N2a cells were transfected with PrP^C^ and labelled with [^35^S] methionine for 1 hour. After labelling cells were either analysed directly (Pulse), or incubated in fresh medium for additional 4 h (Chase). When indicated, mPPIg5 was present during the starving, labelling and chase periods (final concentration 20 µg/ml). PrP was immunoprecipitated by using the monoclonal anti-PrP antibody 3F4 and analysed by SDS-PAGE and autoradiography. Apparent molecular mass based on migration of protein standards is indicated for 16, 22, and 36 kDa.

A possible explanation for mPPIg5′s ability to inhibit the conversion of PrP^C^ to PrP^Sc^ is that mPPIg5 interferes with the biogenesis or subcellular localization of the PrP^C^ protein. mPPIg5 may prevent PrP^C^ from localizing in lipid rafts at the plasma membrane and thus prevent its conversion to PrP^Sc^ in a similar manner to which suramin works for example [8]. To examine this possibility, N2a#58 cells grown on coverslips were treated with mPPIg5 or suramin and PrP^C^ localization examined via confocal microscopy (Figure 7A). Non-permeabilized cells were used to specifically examine PrP^C^ levels at the plasma membrane whilst permeabilized cells were used to detect all PrP^C^ within the cell. The results illustrate that mPPIg5 treatment has no effect on the localization of PrP^C^, which is still predominantly found at the plasma membrane. In agreement with previous results, suramin treatment of cells inhibited PrP^C^ localization at the plasma membrane and instead caused PrP^C^ aggregation in what appears to be a post-ER/Golgi compartment [8].

To confirm the confocal microscopy result, phosphatidylinositol-specific phospholipase C (PIPLC) was utilised. mPPIg5 and mock treated N2a#58 cells were incubated with PIPLC, and following this the cell media was collected and treated cells lysed. The media was ethanol precipitated to obtain all protein. Both this and the cellular lysate were then examined for PrP^C^ content by immunoblot (Figure 7B). The cellular lysate for both the control and mPPIg5 treated cells exhibited a significant drop in PrP^C^ content following PIPLC treatment indicating PrP^C^ was removed from the plasma membrane. This PrP^C^ was detectable in the media, though at a lower level than expected. This discrepancy can be explained by a diminished immunoblot signal from PrP^C^ cleaved by PIPLC [29]. There was no detectable difference between the amount of PrP^C^ released from the plasma membrane in the control and mPPIg5 treated cells indicating that mPPIg5 had no effect on PrP^C^ localization.

To ensure PrP^C^ synthesis was not affected by mPPIg5 treatment, N2a cells were pulse radio labelled with [^35^S] methionine in the presence or absence of mPPIg5 and the synthesis of PrP^C^ monitored (Figure 7C). mPPIg5 treated cells produced an equal amount of PrP^C^ as the control cells indicating mPPIg5 has no effect on PrP^C^ synthesis.

## Discussion

Maltose modified PPI (mPPI) dendrimers are a relatively new class of anti-prion compounds with immense potential due to their low cytotoxicity [14] and ability to cross the blood-to-brain barrier [30]. In this study we developed and utilised novel forms of the scrapie cell assay to demonstrate that the maltose modified PPI dendrimer, mPPIg5, is a potent anti-prion drug which inhibits the conversion of PrP^C^ to PrP^Sc^. In addition, we demonstrate that mPPIg5 does not interfere with the biogenesis or processing of the regular PrP isoform, indicating that modified dendrimers possess significant potential as anti-prion therapeutics and as agents for investigating the nature of prion diseases.

To deduce mPPIg5′s intracellular mode of action, a novel assay called the N terminal specific SCA (nSCA) was developed. The nSCA was used to demonstrate that mPPIg5 inhibits the conversion of PrP^C^ to PrP^Sc^ (Figure 6). This is novel as previous studies have suggested that dendrimers which eliminate PrP^Sc^ from cells achieve their effect by enhancing the degradation of PrP^Sc^ in acidic endosomes/lysosomes [6], [7]. This working hypothesis was based on three observations:

1. The alkalising lysomotrophic agent chloroquine, inhibited the elimination of prions by dendrimers (PEI) [6].
2. Confocal microscopy showed dendrimers (PPI) localizing in lysosomes [7].
3. The *in vitro* elimination of protease resistant PrP^Sc^ by dendrimers (PEI and PPI) was greatest at an acidic pH [6], [7].

Collectively, these findings argue that dendrimers with anti-prion activity achieve their effect by increasing the degradation of PrP^Sc^. However when examined individually, each of the arguments quickly becomes less robust. For example, chloroquine has multiple effects on cells in addition to raising its lysosomal pH such as altering protein trafficking and endocytosis [31], [32]. In addition, NH_4_Cl, another alkalising lysomotrophic agent, does not inhibit the effect of PEI dendrimers [6] suggesting alkalisation of acidic compartments alone is not sufficient. The finding that the *in vitro* elimination of protease resistant PrP^Sc^ by dendrimers was greatest at an acidic pH is also not conclusive as the *in vitro* anti-prion action of dendrimers appears to involve the denaturation of PrP^Sc^ and so an acidic pH enhancing this is not surprising. In addition, an acidic pH is not necessary for the *in vitro* anti-prion effect of dendrimers, it just optimizes it, and so an acidic environment intracellularly cannot be automatically assumed (unpublished data). Finally, the fact that PPI dendrimers localize predominantly in lysosomes is not surprising as most endocytosed materials will end up there. Hence the argument for various dendrimers eliminating prions by increasing their lysosomal degradation is less robust than first impressions would lead one to believe. That said, the ability of numerous dendrimers to render PrP^Sc^ protease sensitive *in vitro* does suggest that dendrimer induced intracellular degradation of PrP^Sc^ is quite possible. The approach employed in this study makes no presumptions on whether or not mPPIg5 interferes with the degradation of PrP^Sc^. It solely examines the issue of whether or not the conversion of PrP^C^ to PrP^Sc^ is being inhibited. For drugs such as STI571 which do not inhibit the conversion process but clearly eliminate prions from infected cells, the nSCA can be used to determine that they must work by increasing degradation through a process of elimination. However the nSCA system cannot be used to tell whether a drug works by both inhibiting conversion and increasing degradation of PrP^Sc^ or by solely inhibiting the conversion of PrP^C^ to PrP^Sc^. Hence it is quite possible that mPPIg5 is able to eliminate prions from a cell by both inhibiting the conversion of PrP^C^ to misfolded forms and increasing the degradation of PrP^Sc^ as *in vitro* experiments suggest.

Evidence for other dendrimers inhibiting the conversion of PrP^C^ to PrP^Sc^ is indirectly supplied from previous studies. An investigation with permanently charged quaternized dendrimers showed that versions of these dendrimers which demonstrated no activity against prions *in vitro*, are capable of eliminating prions intracellularly and inhibiting PrP aggregate formation in PMCA experiments. This suggested to the authors that something other than dendrimer induced degradation of prions may be taking place such as inhibition of PrP^C^ to PrP^Sc^ conversion [12]. In this study we demonstrated a similar result whereby mPPIg5 was not effective against 22L in an *in vitro* setting but highly effective in an intracellular setting (Figure 3). Additionally, experiments with the PAMAM generation 7 (PAMAMg7) dendrimer have demonstrated that this polyamine causes a dramatic accumulation of an insoluble, protease-sensitive PrP aggregate, believed to be produced directly from PrP^C^. It is possible that this aggregate inhibits the formation of PrP^Sc^ by sequestering PrP^C^ [11]. Our study did not reveal any evidence of such non-infectious PrP aggregates following treatment with mPPIg5 suggesting this may be an effect specific to PAMAM or perhaps even high generation dendrimers.

mPPIg5 has no effect on PrP^C^ synthesis and localization as demonstrated by confocal microscopy, pulse chase and PIPLC experiments. Since mPPIg5 has no discernible effect on PrP^C^, how then does it inhibit the conversion of PrP^C^ → PrP^Sc^? There are a number of possibilities. mPPIg5 may competitively inhibit the interaction between PrP^C^ and PrP^Sc^. Or it may interfere with components of PrP^Sc^ formation which have not yet been identified such as short-lived PrP^C^ → PrP^Sc^ intermediates or potential co-factors like RNA which are necessary for PrP^Sc^ formation [33]. Alternatively PrP^Sc^ trafficking may be interfered with to the extent that it is no longer available for the conversion of PrP^C^.

Perhaps the most likely explanation is that mPPIg5 alters the structure of PrP^Sc^ to such an extent that it is no longer capable of initiating the misfolding of PrP^C^. Evidence for dendrimers effecting the structure of PrP^Sc^ comes from *in vitro* studies with prion infected brain homogenate, demonstrating that various dendrimers appear to denature PrP^Sc^ molecules and render them susceptible to proteolysis [6], [7]. Additionally, Fourier transform infrared spectroscopy and electron microscopy studies have demonstrated that purified prions treated with PPI dendrimers lose β-sheet structure and become disaggregated [7]. Dendrimers altering the confirmation of the PrP^Sc^ molecule can thus serve as an explanation for both the *in vitro* and intracellular activity of all dendrimers. One could envisage dendrimers interacting with the PrP^Sc^ molecule (both *in vitro* and intracellularly) and altering its structure in a process that is governed by the original confirmation of the PrP^Sc^ molecule (influenced by the type of prion strain) and on the chemical nature of the dendrimer (dependant on a combination of the dendrimers size, structure, charge and density of reactive surface groups). In some instances, the interaction will alter the PrP^Sc^ structure to the extent that it becomes susceptible to proteolysis, both in an *in vitro* and intracellular setting. In other cases the interaction will not be sufficient to render PrP^Sc^ susceptible to proteolysis but it will alter the PrP^Sc^ molecule to the extent that it can no longer convert PrP^C^ to the misfolded form. This would explain how some dendrimers apparently fail to reduce protease resistant PrP^Sc^ *in vitro* but can be effective intracellularly. Additionally, such a scenario would allow a dendrimer to eliminate prions intracellularly by both increasing their degradation as the original experiments in this area suggest [6], [7], [34] and/or eliminate prions by inhibiting the conversion of PrP^C^ to PrP^Sc^ as some of the more recent studies in this area, including our own, suggest [11], [12].

Dendrimers affecting the structure of misfolded PrP may also explain how numerous dendrimers (including mPPI) inhibit the fibrilization of PrP peptides *in vitro* [13], [35]–[40]. This ability is believed to be due to dendrimers increasing the fibril breakage rate or blocking the end of growing fibrils [41], [42]. Both of these scenarios are possible in an intracellular setting. However dendrimers blocking the end of growing fibrils cannot explain the ability of various dendrimers to reduce PrP^Sc^ in prion infected brain homogenate [6], [7], [14], [34]. Dendrimers increasing the fibril breakage rate on the other hand can explain this ability and again may be linked back to specific dendrimers having an effect on the structure of misfolded PrP. Hence an ability to alter the structure of PrP^Sc^ can explain the various anti-prion activities of dendrimers in an intracellular and *in vitro* setting. Whilst it is possible that different dendrimers may achieve their anti-prion effect through a variety of mechanisms, Occam′s razor dictates that a single explanation is the most likely. Additional experiments are necessary before this hypothesis could be accepted. What this study demonstrates is that mPPIg5 inhibits the conversion of PrP^C^ to PrP^Sc^. Understanding the basis of the anti-prion activity of other dendrimers remains an exciting challenge.

This study also involved the development of the modified SCA (mSCA) as a high throughput and fully quantitative system for monitoring the elimination of prions in infected cells over time. The traditional method for assessing a drug's anti-prion effect in an intracellular setting relies on the PK digestion and immunoblotting of samples followed by densitometry to quantify the result. Such immunoblot based assays are labour intensive and the densitometry involved generates, at best, a semi-quantitative result. The mSCA is a marked improvement over these traditional methods. It offers a number of advantages in speed and throughput for the assessment of the efficacy of anti-prion drugs and is suitable for the mass screening of compounds.

In conclusion, this study highlights how the mSCA and nSCA represent exciting new tools for investigating the efficacy and mode of action of novel anti-prion compounds. These assays were utilised to demonstrate that mPPIg5 is an effective anti-prion compound capable of inhibiting the conversion of PrP^C^ to PrP^Sc^. Understanding how a drug works is a vital component in maximising its performance. By establishing that mPPIg5 interferes with the conversion of PrP^C^ to PrP^Sc^, this study will help determine what other drugs may enhance this effect. The low cytotoxicity of mPPIg5 and the recently demonstrated ability of maltotriose dendrimers to cross the blood to brain barrier [30] suggests that mPPIg5 is one of the most promising anti-prion therapeutics currently available and worthy of further investigation.

## Supporting Information

Figure S1 **ELISPOT after mSCA and sSCA.** Elispot well after the mSCA and the sSCA of N2a and ScN2a cells. The spots visible on the ELISPOT membrane reflect cells containing PrP^Sc^. The spots produced by the mSCA are sharper and more intense than those produced by sSCA. All images were captured in maximum focus and not subsequently altered in any way.
(TIF)

Figure S2 **Optimum mPPIg5 treatment concentration.** 22LN2a#58 cells were treated with increasing concentrations of mPPIg5 for **A**) 26 hours or **B**) 72 hours before analysis by mSCA (20,000 cells/well). The optimum concentration of mPPIg5 for PrP^Sc^ elimination was found to be 10–20 µg/ml, which is similar to that calculated for the elimination of PrP^Sc^ in RML infected cells 14). Error bars represent SD; n = 3.
(TIF)

Figure S3 **Preparation and validation of PrP^27–30^.** **A**) To generate PrP^27–30^ free of any traces of FL PrP^Sc^ an innovative method was employed. ScN2a cells were lysed in a minimum detergent lysis buffer to reduce the toxicity of the extract. Following this, PrP^Sc^ present in the lysate was reduced to PrP^27–30^ using an extended PK digest and wash protocol. To ensure that the PrP^27–30^ produced was N terminally cleaved an immunoblot was performed with monoclonal antibody SAF32 (specific for the N terminal of PrP; lane 1 and 2) and SAF83 (specific for the protease resistant core of PrP^27–30^; lane 3 and 4). Samples in lane 1 and 3 are non-protease treated controls whilst samples in lane 2 and 4 were PK treated for 2 hours. Apparent molecular mass based on migration of protein standards is indicated for 17, 25, and 30 kDa. **B**) The infectivity of the PrP^27–30^ produced (and hence its similarity to endogenous PrP^27–30^) was examined by the scrapie cell assay. A highly susceptible subclone of N2a cells (N2a29) and a subclone resistant to prion infection (N2a6) created in our laboratory were seeded onto a 96 well TC plate and inoculated with various dilutions of PrP^27–30^ purified from ScN2a cells. The cells were passaged four times to dilute the original inoculate and then examined via mSCA for infected N2a cells. A 1/10 dilution of PrP^27–30^ proved toxic to the cells. N2a cells resistant to prion infection (N2a6) were used to show that none of the original PrP^27–30^ inoculate was detectable after 4 passages. Error bars represent SD; n = 3 (technical, not biological repeats). The immunoblotting and infectivity study demonstrated that the PrP^27–30^ prepared from ScN2a cells lacked the N terminal but retained its protease resistant core and the ability to infect susceptible cells.
(TIF)
