# Supplementary figures and images for: Anti-Prion Drug mPPIg5 Inhibits PrPC Conversion to PrPSc
Source: PLoS One. 2013 Jan 28;8(1):e55282. doi: 10.1371/journal.pone.0055282 (PMC3557256; doi:10.1371/journal.pone.0055282)

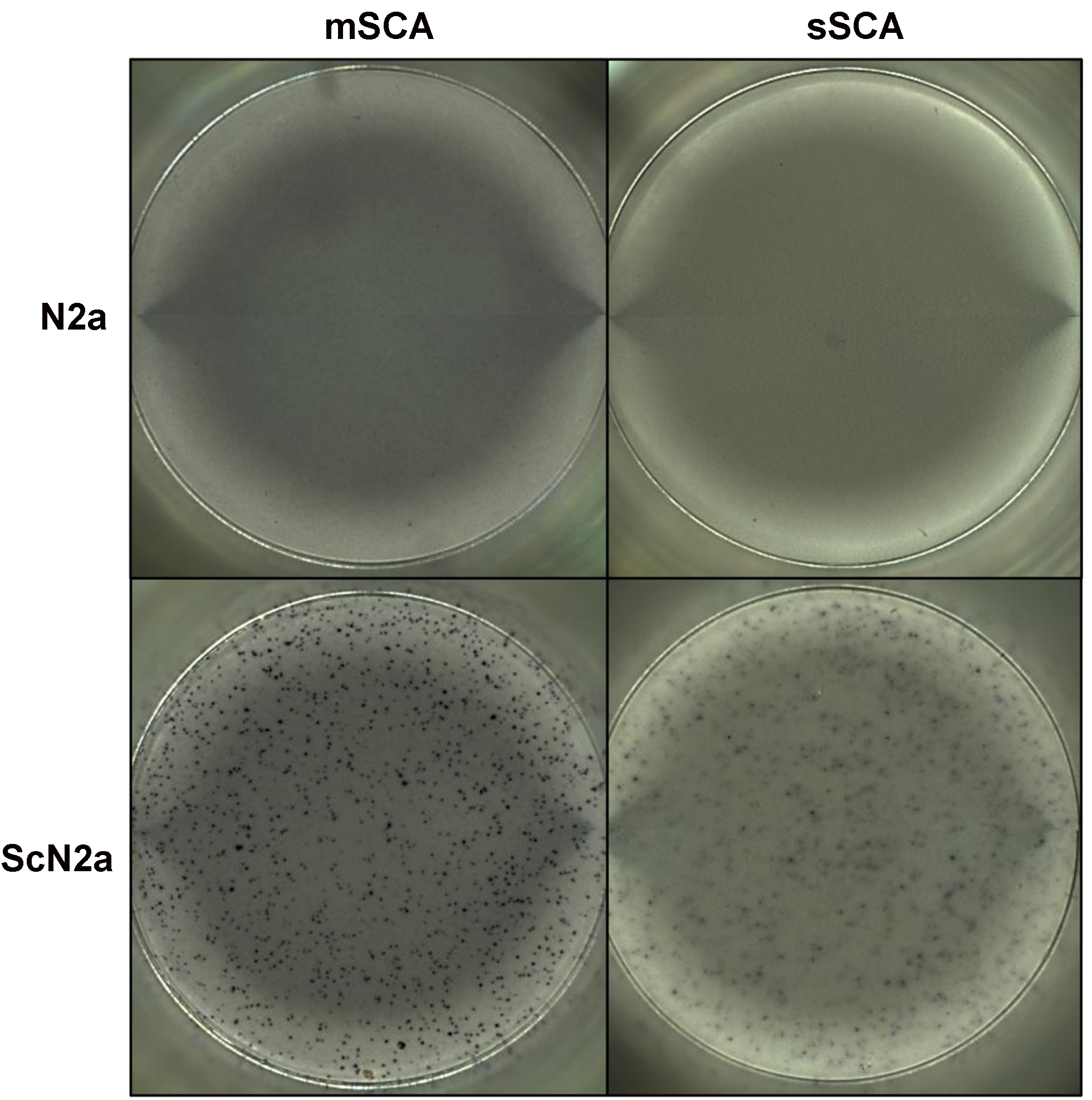

Supplement: Figure S1 — ELISPOT after mSCA and sSCA. Elispot well after the mSCA and the sSCA of N2a and ScN2a cells. The spots visible on the ELISPOT membrane reflect cells containing PrPSc. The spots produced by the mSCA are sharper and more intense than those produced by sSCA. All images were captured in maximum focus and not subsequently altered in any way. (TIF) [file pone.0055282.s001.tif]

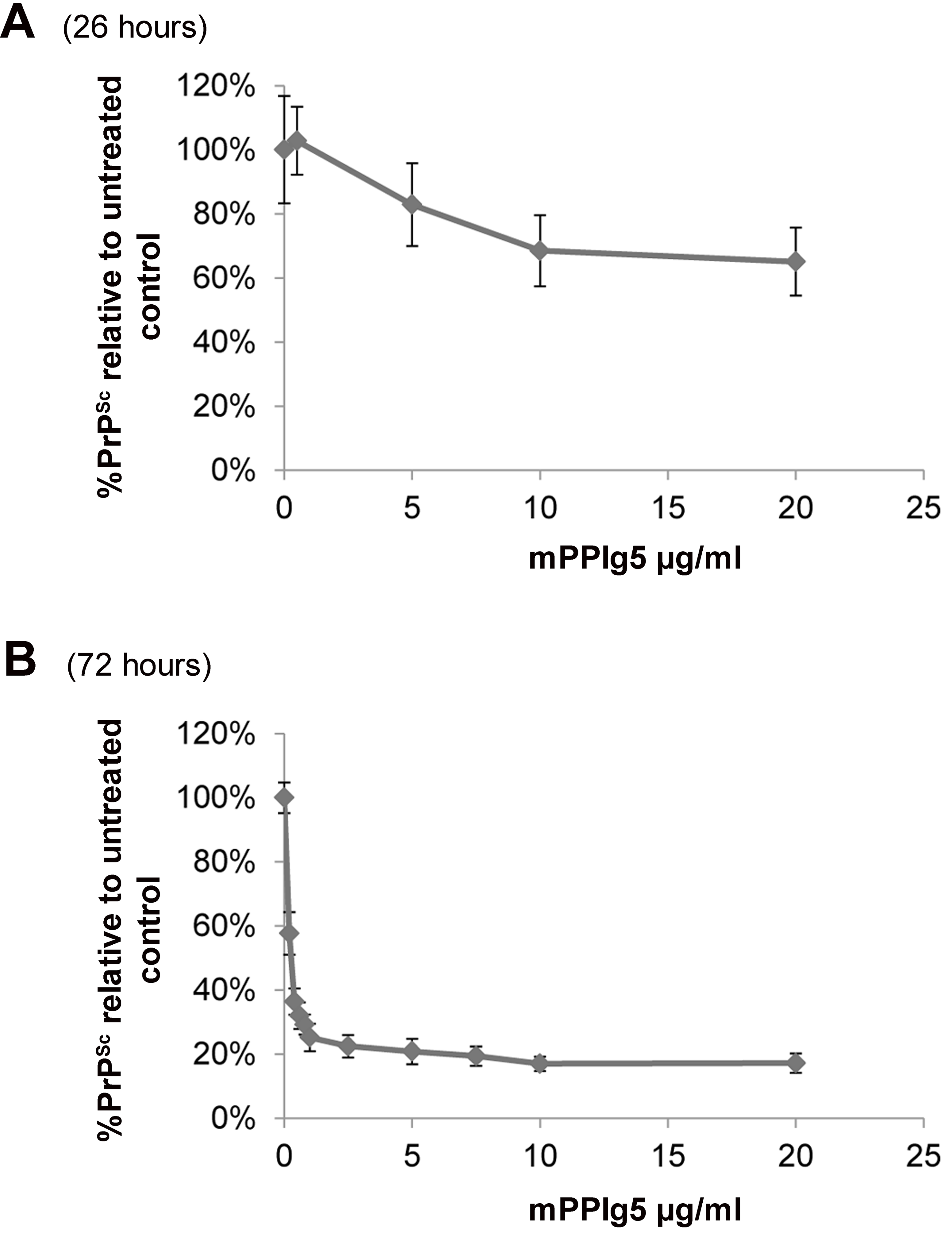

Supplement: Figure S2 — Optimum mPPIg5 treatment concentration. 22LN2a#58 cells were treated with increasing concentrations of mPPIg5 for A) 26 hours or B) 72 hours before analysis by mSCA (20,000 cells/well). The optimum concentration of mPPIg5 for PrPSc elimination was found to be 10–20 µg/ml, which is similar to that calculated for the elimination of PrPSc in RML infected cells 14). Error bars represent SD; n = 3. (TIF) [file pone.0055282.s002.tif]

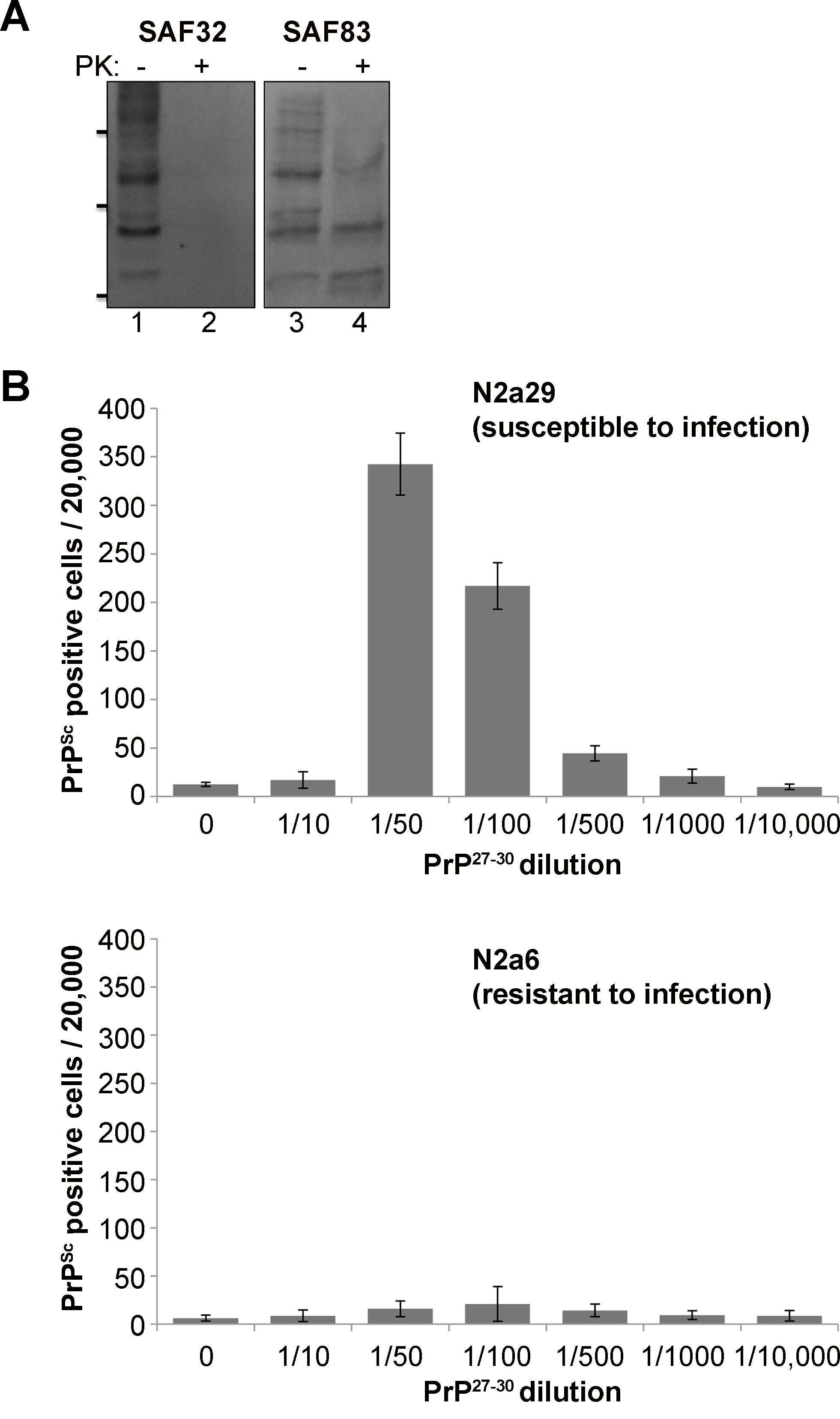

Supplement: Figure S3 — Preparation and validation of PrP27–30. A) To generate PrP27–30 free of any traces of FL PrPSc an innovative method was employed. ScN2a cells were lysed in a minimum detergent lysis buffer to reduce the toxicity of the extract. Following this, PrPSc present in the lysate was reduced to PrP27–30 using an extended PK digest and wash protocol. To ensure that the PrP27–30 produced was N terminally cleaved an immunoblot was performed with monoclonal antibody SAF32 (specific for the N terminal of PrP; lane 1 and 2) and SAF83 (specific for the protease resistant core of PrP27–30; lane 3 and 4). Samples in lane 1 and 3 are non-protease treated controls whilst samples in lane 2 and 4 were PK treated for 2 hours. Apparent molecular mass based on migration of protein standards is indicated for 17, 25, and 30 kDa. B) The infectivity of the PrP27–30 produced (and hence its similarity to endogenous PrP27–30) was examined by the scrapie cell assay. A highly susceptible subclone of N2a cells (N2a29) and a subclone resistant to prion infection (N2a6) created in our laboratory were seeded onto a 96 well TC plate and inoculated with various dilutions of PrP27–30 purified from ScN2a cells. The cells were passaged four times to dilute the original inoculate and then examined via mSCA for infected N2a cells. A 1/10 dilution of PrP27–30 proved toxic to the cells. N2a cells resistant to prion infection (N2a6) were used to show that none of the original PrP27–30 inoculate was detectable after 4 passages. Error bars represent SD; n = 3 (technical, not biological repeats). The immunoblotting and infectivity study demonstrated that the PrP27–30 prepared from ScN2a cells lacked the N terminal but retained its protease resistant core and the ability to infect susceptible cells. (TIF) [file pone.0055282.s003.tif]
